# Effect of intraradicular reinforcement strategies on the fracture strength of endodontically treated anterior teeth with overflared canals

**DOI:** 10.4317/jced.58862

**Published:** 2022-01-01

**Authors:** Mehdi Biabani-Sarand, Mahmoud Bahari, Mehdi Abed-kahnamoui, Mohammad-Esmaeel Ebrahimi-Chaharom, Shahriar Shahi

**Affiliations:** 1 Undergarduate student, Dental Faculty, Tabriz University of Medical Sciences, Tabriz, Iran; 2 DDS, MS. Dental and Periodontal Research Center, Dental Faculty, Tabriz University of Medical Sciences, Tabriz, Iran; 3 DDS, MS. Department of Operative Dentistry, Dental Faculty, Tabriz University of Medical Sciences, Tabriz, Iran; 4 DDS, MS. Department of Endodontics, Dental Faculty, Tabriz University of Medical Sciences, Tabriz, Iran

## Abstract

**Background:**

This study is done to evaluate the effect of different reinforcement techniques of overflared root canals with fiber posts on the fracture resistance of endodontically treated anterior teeth. It also introduces a new technique for simultaneous reinforcement and cementation of fiber posts using dual-cured core build-up composite resin.

**Material and Methods:**

The crowns of 48 single-rooted maxillary anterior teeth were cut and randomly divided into 4 groups (n=12) based on root reinforcement techniques after root canal treatment and post space preparation: Group 1: self-adhesive resin cement (SARC), Group2: dual-cure composite resin (DCC), Group3: Composite resin reinforcement (CRR), Group 4: Direct anatomical post (DAP). Then a core was made for the roots. The periodontal ligament was simulated using a thin layer of PVC impression material. A universal testing machine applied a compressive force to the lingual surface at an angle of 135 ° and 1 mm/min speed. Data were analyzed using One-Way ANOVA and a post-hoc Tukey test. Fracture patterns were analyzed using the Chi-square test (*p*<0.05).

**Results:**

The difference between the control and DAP group was statistically significant (*p*=0.01), but there were no significant differences between other groups (*p*>0.05). Regarding fracture patterns, there were no significant differences between groups.

**Conclusions:**

All reinforcement techniques increased the fracture strength compared to the control group. However, DAP was the only group that had a statistically significant difference. CRR and DCC presented intermediate values without significant differences.

** Key words:**Fiber post, endodontically treated teeth, root canals, reinforcement, fracture strength.

## Introduction

Endodontically treated teeth with extensive tissue loss usually need a post to retain a core and final restoration. Nowadays, fiber posts have become more common due to good functional and esthetic results, lower cost, and treatment time. Also, they do not corrode like metal posts and can be easily removed if endodontic retreatment is needed. An important characteristic of fiber posts is their modulus of elasticity, similar to that of dentin. As a result, the fiber posts are more flexible under pressure and have a better stress distribution, which reduces the risk of root fractures (1,2).

Overflared root canals can be caused by Extensive caries, previous restoration with large diameter post and cores, aggressive endodontic treatment, incomplete root formation, traumatic dental injuries, internal resorption, and even due to the oval shape of the canals (3). Cast metal posts and glass fiber posts are two techniques to repair these damaged teeth. A systematic review and meta-analysis showed a higher survival rate for fiber post in the medium term (1), but controversies exist (4,5). Prefabricated fiber posts are not fully adaptable with root canal anatomy and canal walls. So, using a single fiber post in overflared canals may result in poor adaptation, core mutability, increase in cement thickness, and reduced mechanical properties (6,7).

On the other hand, a large amount of resin cement is needed to fill the gap. In this case, bubbles form inside the cement and may cause debonding, which is the most common problem with this type of post (8,9). Also, these types of cement have high polymerization shrinkage, which increases stress concentration in the interface area. The use of accessory posts with the main fiber post does not significantly reduce the thickness of the cement. On the other hand, due to the increased interfaces between the reinforcing components and dentin may complicate the adhesive effect of this technique (8,10,11).

Recently, overflared canal problems can be overcome by direct (DAP) and indirect (IAP) reconstruction of anatomical fiber posts. According to the literature, this method reduces the thickness of the resin cement, increases its durability and mechanical properties (12-16). Clavijo *et al*. demonstrated that direct and indirect anatomic posts could be an alternative to cast metal post-and-cores during the treatment of overflared root canals (15). Some studies have recently proposed another method for relining and reinforcing weakened root canal walls with composite resin before fiber post cementation (10,17,18). Gomes *et al*. reported that this technique is as efficient as DAP and IAP in fracture resistance (10).

Resin cements have a similar composition to composite resin but have a lower filler content. There is a relationship between filler content and physical and mechanical properties. Materials with higher filler content have more hardness, stiffness, durability, compressive and flexural strength. They also have less shrinkage during polymerization. The lower viscosity of resin cements than composite resins causes higher shrinkage stress and increases the probability of debonding and microleakage (19). Therefore, this study introduces the new technique of simultaneous reinforcement and cementation of fiber posts to overflared canals using dual-cured core build-up composite resin materials, which is a one-step method and compares its fracture strength with previously mentioned methods. This one-step method is simpler, more practical, and needs less chair-time. The null hypothesis studied was that fracture strength of anterior teeth is similar to that of overflared canals using different reinforcement techniques.

## Material and Methods

Forty-eight single-rooted maxillary anterior teeth that were extracted for periodontal reasons and have healthy roots were selected. The root surfaces were cleaned and kept in 0.5% chloramine T solution until use. The working length and morphology of canals were determined by radiography. Teeth with caries or root fractures, calcification within the root canal, severely curved roots, working length (WL) out of the range of 14±1 mm, or a history of endodontic or previous post-treatment were excluded from the study.

A diamond disk (Diamant GmbH, D&Z, Berlin, Germany) cut the crowns in a low-speed straight handpiece (Kavo Dental GmbH, Bismarckring, 39D-88400 Biberach/ Riß. Germany) under continuous water spray. The crown down technique was used to prepare the canal space. Canals obturated with gutta-percha points (Dia Dent, Chungcheongbuk, Korea) and AH26 sealer (Dentsply, DeTrey, Konstanz, Germany) by lateral condensation technique up to one mm left to the WL. Radiographic images were taken to ensure the quality of obturations. The samples were placed in an environment with 100% humidity for 72 hours. Then 10 mm of gutta-percha was emptied to prepare the post space. First, gutta-percha was removed by gates glidden drills (Mani, Tochigi, Japan). A low-speed peesoreamers (Mani, Tochigi, Japan) then prepared the canals. The post space length was 10 mm, and at least 4 mm of gutta-percha remained at the end of the canal.

To simulate flared root canals, a 2.5 mm diameter diamond bur (Teeskavan, Tehran, Iran) was used in a low-speed handpiece to a depth of 10 mm, also an approximately 1 mm root dentin wall thickness was maintained. Then, the canals were irrigated with normal saline and dried by paper points (DiaDent, Chungcheongbuk, Korea). Fiber Post No. 2 was used in all canals. According to the manufacturer’s instructions, Exacto Glass fiber posts (Angelus, Londrina, PR, Brazil) were tested inside the canals and then cleaned with alcohol. Finally, the roots were randomly divided into 4 groups (n=12) based on root reinforcement techniques.

First group: Self-adhesive resin cement (SARC)

Root canal walls were impregnated with All-Bond Universal Adhesive (Bisco, Schaumburg, IL, USA) for 15 seconds and gently air-dried for 10 seconds. According to the manufacturer’s instructions, the fiber posts were impregnated with a silane coupling agent (Bisco, Schaumburg, IL, USA) and then cemented with G-CEM LinkAce self-adhesive resin (GC Corp., Tokyo, Japan) cement into the root canal. Cement inserted into canals by spiral lentulo and fiber post covered with resin cement placed inside the canal with controlled finger pressure and the excess resin removed using paper points. Then, using a Demetron A2 LED light curing device (Kerr, Orange, CA, USA) with a light intensity of 1000 mW/cm2, the light-curing operation was performed for 30 seconds while the tip of the device was in contact with the end of the post.

Second group: Dual-cured core build-up composite resin (DCC) 

First, the root canal walls were impregnated with All-Bond Universal adhesive (Bisco, Schaumburg, IL, USA) for 15 seconds and gently air-dried for 10 seconds. Luxacore Z (DMG, Hamburg, Germany), A dual-cured core build-up composite resin, was placed inside the root canal using spiral lentulo. Then silane (Bisco, Schaumburg, IL, USA) impregnated fiber post was immediately placed inside the canal. The light-curing process was performed similar to group 1.

Third group: Composite resin reinforcement (CRR)

The root canals were impregnated with All-Bond Universal adhesive (Bisco, Schaumburg, IL, USA) for 15 seconds and gently air-dried for 10 seconds. Opus bulk-fill APS composite resin (FGM Dental Group, Zona Industrial Norte, Joinville – SC, Brazil) was then placed inside the root canal. The fiber post has already been lubricated with a hydro-soluble gel (KY, Johnson & Johnson, Sa˜oJose ose dos Campos, SP, Brazil) placed inside the canal. The light-curing process was performed in contact with the top of the translucent fiber post for 60 seconds. The post was then removed, and the composite resin was light-cured inside the root canal for 40 seconds by contacting the tip of the light cure device with the root canal. The post and canal were washed and air-dried. Then the post was cemented into the canal similar to the first group.

 In the fourth group (DAP), a hydro-soluble gel (KY, Johnson & Johnson, Sa˜oJose ose dos Campos, SP, Brazil) was first used as a lubricant inside the root canal. The post was impregnated with silane (Bisco, Schaumburg, IL, USA), and then a layer of non-polymerized Opus bulk-fill APS composite resin (FGM Dental Group, Zona Industrial Norte, Joinville – SC, Brazil) was placed on it and taken into the canal. Then it was cured for five seconds with the light intensity of 1000 mW/cm2 with an LED light-curing device (Demetron A2, Kerr, Orange, CA, USA), and the set was then removed from the canal, and another curing process was done outside of the canal for 20 seconds on each side. The post and canal were washed, and the DAP was cemented into the canal, similar to the first group.

The samples were kept at 100% humidity for 24 hours. A core was then prepared for the roots of all four groups using Opus bulk-fill APS composite resin (FGM Dental Group, Zona Industrial Norte, Joinville – SC, Brazil). To simulate the periodontal ligament, the roots were covered with 0.3 mm thick wax and embedded up to 3 mm below the CEJ in a plastic mold filled with an autopolymerized acrylic resin. After polymerization, the teeth were removed and cleaned from waxes. The roots were then re-inserted into acrylic resin blocks filled with bonasil low viscosity vinyl polysiloxane impression material (DMP, Markopoulo Industrial Zone, Greece). Samples were placed inside the universal testing machine (Hounsfield test equipment, HSKS model, surrey, UK) for fracture resistance tests. A compressive force was applied to the lingual surface (2 mm below the incisal edge) at an angle of 135 ° to the tooth’s longitudinal axis at a 1 mm/min cross-head speed. This force continued until fracture occurred. Fracture resistance values were recorded in Newtons. Fracture patterns were classified as repairable and non-repairable ones according to the location of the fracture line.

Fracture strength data were analyzed using One-Way ANOVA and a post-hoc Tukey test. Fracture patterns were analyzed using the Chi-square test. The significance level was set at *p*<0.05.

## Results

**Figure 1:**
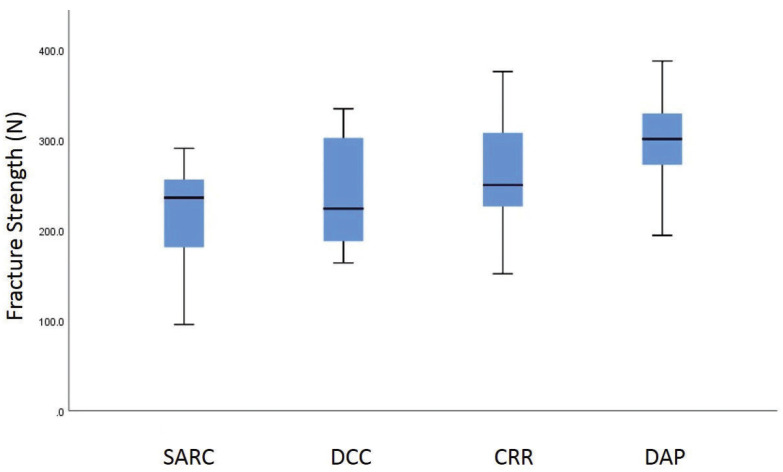
Error-bar graph of fracture strength values (Mpa).

Table 1 presents mean ± standard deviations of fracture strength values. Shapiro-wilk test showed that data has a normal distribution (*p*=0.14). One-way ANOVA revealed a statistically significant difference between groups (*p*=0.02). Post-hoc Tukey test demonstrated that the difference between the control and DAP group was statistically significant (*p*=0.01). However, there were no statistically significant differences between the other groups (*p*>0.05). Figure 1 shows the Error-bar graph of fracture strength values.

**Table 1:**
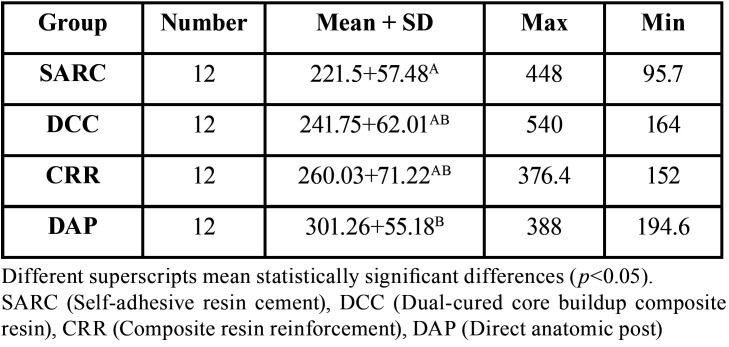
Descriptive statistics for fracture strength values (Mean ± SD) (N).

**Figure 2:**
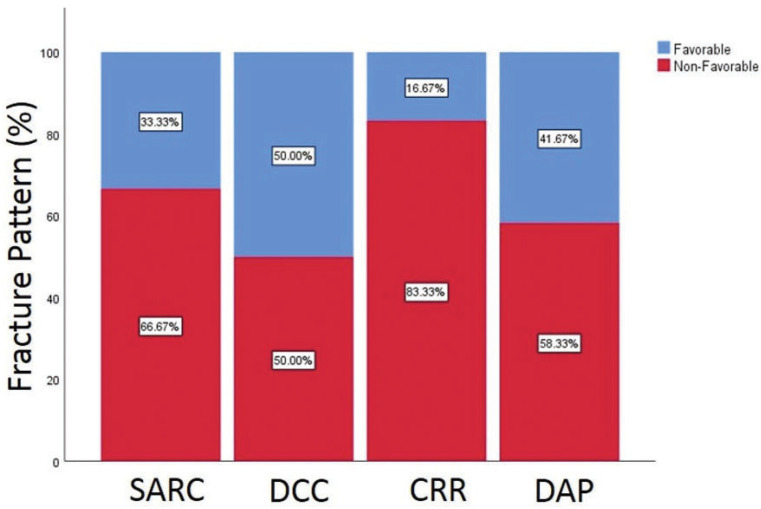
Bar chart of percentage of fracture patterns.

Regarding fracture patterns, 33.33% of the teeth in the control group, 50% in the DCC group, 16.67% in the CRR group, and 41.67% in the DAP group were repairable. According to the Chi-square test, there were no significant differences between groups (*p*=0.44). Figure 2 shows a bar chart of the percentage of fracture patterns.

## Discussion

Restoration of Endodontically treated teeth with overflared root canals is still a challenge due to the mismatch between the walls of the wide canals and the diameter of the prefabricated fiber posts, which results in a large thickness of cement (16,20). Bubbles, cracks, and gaps are more likely to form in thick cement, creating a concentrated stress area and reducing bond strength. Several techniques have been proposed to solve this problem, including restoration of the root canal with composite resin to decrease canal width, direct and indirect anatomical shaping of fiber posts with a composite resin, and the use of accessory posts (13,14,18,21). Another technique proposed in the present study uses dual-cured core build-up composite resin instead of self-adhesive resin cement for simultaneous reinforcement and cementation of fiber posts to overflared canals.

In this study, the difference in fracture resistance between the groups was statistically significant. Therefore, the proposed null hypothesis was rejected. DAP was the only experimental group that had significantly higher resistance than the control group. Other groups have intermediate fracture strength between the two groups, and there was no significant difference.

In vitro measurement of the difference between the fracture resistance of reinforced teeth by different methods provide an estimate of the load-bearing capacity of the reinforcing systems and a basis for clinical studies necessary for decision making (22). To standardize the samples, teeth of similar size and shape extracted from a specific age group were used in this study. In this study, acrylic resin molds were used because their modulus of elasticity is similar to human bone. Also, to simulate the surface of the crestal bone as much as possible, the embedded roots were exposed 3 mm outside the acrylic molds (23).

In addition, the periodontal ligament was simulated using a thin layer of PVC impression material to further simulate the clinical condition and accurately assess the fracture resistance. In studies that do not perform this simulation, unrealistic force values may be obtained that affect the failure pattern of the specimens and affect the comparison of groups (23). Another important point is the contact angle of the applied force with the sample, which can greatly affect the fracture resistance. The more force parallel to the longitudinal axis of the tooth, the greater the fracture strength (24).

In this study, all reinforcement strategies performed better than the control group; because the resin composite absorbs and distributes forces more uniformly and may increase the fracture strength of the teeth restored with post and core. However, only in the DAP group, the fracture strength was significantly higher. In CRR and DCC groups, there was no significant difference between them despite higher fracture resistance than the control group. In the CRR group, the lack of adequate curing of the bulkfill resin composite in the deepest parts of the canal may be responsible for this. Although the study used transparent posts that can transmit light to deeper areas, they may not be enough for sufficient polymerization of the composite resin in deeper parts of the canal alone (25-27).

Studies that have examined the effectiveness of different methods of relining and reinforcing in overflared root canals have reported different results and are difficult to compare due to their different methods (13,15,25,26,28,29). In a systematic review and meta-analysis, Silva *et al*. found no significant difference in fracture resistance of fiber posts reinforced with auxiliary fiber posts or composite resin and non-reinforced posts (12). The relining procedure of fiber post increases the cost of materials and clinical chair-time, so most clinicians may refuse to use these techniques due to the lack of significant improvement (12). Therefore, this study can be important because of the significant differences in fracture resistance between the experimental groups and the non-reinforced group.

Goncalves *et al*., reported that although reinforcement of root canal walls with different composite resins, such as the CRR group in this study, increased fracture resistance compared to weakened root canals restored with post and core but there was no significant difference with the control group without weakened roots that restored with cast post and cores (28). Braz *et al*. used a similar method to reinforce weakened root canals with bulk-fill composite to reduce polymerization shrinkage stress but did not ensure adequate polymerization of the bulk-fill composite resin at apical one-third and suggested the use of dual-cured composite resins (17). In the present study, Luxacore Z dual-cured composite resin, presented for foundation restorations, was used in the DCC group to strengthen canal walls and cement fiber post simultaneously. The fracture resistance of this group was in the range between the control and the DAP group and was not significantly different from them.

According to the study of Belli *et al*., the present study results are remarkable, who used finite element analysis to investigate the effect of different restorative methods on stress distribution by simulating overflared roots of central Incisor. They demonstrated that increasing the thickness of the root walls with composite resin puts less stress on the remaining dentinal tissues. However, still, the total cumulative stress on the dentin was higher than the anatomical post. According to their results, the anatomical post kept the stress inside the post body and transferred less stress to the remaining root tissue(30). Therefore, according to the above mentioned, the use of the anatomical fiber post method in restoring anterior teeth with overflared root canals is preferred. However, performing CRR and especially DCC techniques is less complicated and more practical, especially in the cases of internal resorptions, and root canals with tissue undercuts.

The present study was an *in vitro* study with specific limitations that should caution in generalizing to clinical conditions. For example, the forces applied to the teeth in clinical conditions are repetitive and have different directions that have not been simulated in this study.

## Conclusions

All reinforcement techniques increased fracture strength compared to the control group. However, DAP was the only group that had a statistically significant difference. CRR and DCC presented intermediate values with no significant differences.
